# Profile of Cardiovascular Autonomic Dysfunctions in Breast Cancer Patients

**DOI:** 10.7759/cureus.46773

**Published:** 2023-10-10

**Authors:** Ekta Khandelwal, Sumeet Tripathi, Ashutosh Gupta, Alok Singh

**Affiliations:** 1 Department of Physiology, All India Institute of Medical Sciences, Raipur, Raipur, IND; 2 Department of Physiology, Pt. Jawahar Lal Nehru Memorial Medical College, Raipur, IND; 3 Department of Surgical Oncology, Pt. Jawahar Lal Nehru Memorial Medical College, Raipur, IND; 4 Department of Pharmacology, All India Institute of Medical Sciences, Raipur, Raipur, IND

**Keywords:** cardiovascular, chemotherapy, breast cancer, heart rate variability, autonomic dysfunction

## Abstract

Background

Patients on breast cancer chemotherapy frequently present with signs and symptoms of autonomic dysfunction. Cardiac autonomic dysfunction (CAD) is a significant cause of morbidity and mortality, affecting the quality of life with progressive advancing disease. It is associated with the short survival of breast cancer patients. Therefore, thoroughly assessing cardiovascular autonomic functions is crucial to monitor these patients’ disease prognosis and chemotherapy side effects. The present study evaluated baseline heart rate variability (HRV) and Ewing’s battery of cardiac autonomic reactivity tests in breast cancer patients on chemotherapy to evaluate CAD.

Methodology

This is a case-control study. Autonomic reactivity tests were performed in the autonomic function laboratory, Department of Physiology, All India Institute of Medical Sciences, Raipur. HRV was recorded using a lead II electrocardiogram (ECG) in two groups, namely, controls (n = 25 healthy female volunteers) and cases (n = 25 histologically proven stage I-III breast cancer patients, age 30-65 years, received three cycles of chemotherapy).

Results

Patients on chemotherapy had significantly lower reactivity for the time domain (all parameters) and frequency domain (absolute total power) of HRV compared to age-matched healthy controls. Autonomic reactivity showed significant loss in the patient group.

Conclusions

The sympathetic and parasympathetic parameters showed a significant loss of autonomic functions in the patient group compared to the healthy controls. This may be because of the chemotherapeutic drugs taken by the patients or cancer as the disease per se. As autonomic dysfunction is highly prevalent in patients with cancer and is associated with multiple symptoms, it is essential to study it in the cancer population.

## Introduction

Cardiac autonomic dysfunction (CAD) is a known but unaddressed side effect of cancer chemotherapy. Inside the body, the neural network of the autonomic nervous system is widely distributed, regulating homeostasis. This system can easily be affected by cancer treatment. Patients on chemotherapy frequently present signs and symptoms of autonomic dysfunction [1], such as lower blood pressure, palpitations, tingling, orthostasis, fatigue, gastroparesis, and constipation, which are grossly underdiagnosed. CAD is a critical cause of morbidity and mortality, affecting the quality of life with progressive advancing disease, and is associated with shorter survival of patients with cancer [2]. With timely diagnosis and early interventions, autonomic dysfunction can be treated so that many patients with cancer can benefit [3]. Therefore, a thorough assessment of autonomic functions is crucial for the prognosis of the disease and the side effects of chemotherapy on the cardiovascular autonomic system [4]. It is essential to establish non-invasive methods for regular assessment of autonomic functions for risk stratification and subsequent management [5]. In the present study, we evaluated baseline heart rate variability (HRV) [6] and Ewing’s battery of cardiac autonomic reactivity tests [7] in patients with breast cancer on chemotherapy to evaluate CAD.

## Materials and methods

After obtaining Institutional ethical clearance from All India Institute of Medical Sciences (AIIMS), Raipur, Chhattisgarh, India (approval number: 1376/IEC-AIIMSRPR/2020) and informed written consent from patients, we recruited 33 patients with histologically proven stage I-III breast cancer with age ranging from 30 to 65 years. A total of 25 patients were included without any pre-existing comorbidities such as diabetes, hypertension, or other possible causes of autonomic neuropathy. Patients with Karnofsky’s performance status (KPS) of 80 or more were included [8]. All patients received three cycles of chemotherapy with a combination of regimes, including doxorubicin, paclitaxel, 5-fluorouracil (5-FU), cyclophosphamide, and cisplatin. All patients had average complete blood counts (hemoglobin, total leukocyte count, and platelet count), fasting serum glucose, renal function test (serum urea and creatinine), and liver function test (serum bilirubin, serum glutamic-oxaloacetic transaminase, and serum glutamate-pyruvate transaminase). No patient had any psychiatric or orthopedic disorder. Patients with advanced disease and metastasis were excluded. The age-matched control group (n = 25) consisted of healthy female subjects with no history of chronic illnesses such as diabetes, hypertension, or any neurological disease requiring active treatment.

The study was performed under standardized conditions in the Autonomic Function Laboratory, Department of Physiology, AIIMS, Raipur. A total of 25 patients with breast cancer were recruited from the outpatients of the Department of Oncology, Pt. Jawahar Lal Nehru Memorial Medical College and BR Ambedkar Hospital Raipur based on a complete history, histologically proven cancer, and KPS criteria. Informed written consent was obtained both in English and the local language. Patients were familiarised with the AFT laboratory, and the experimental design and procedure of all reactivity tests, lying to standing (LST), deep breathing test (DBT), Valsalva maneuver (VM), and cold pressor test (CPT) were explained. When patients were comfortable with the procedure, experiments were conducted at an ambient room temperature (25°C-27°C) where external disturbances were minimal between 9:00 a.m. and 11:00 a.m. Patients were instructed not to intake tea, caffeine, or nicotine for 12 hours before the test. The same protocol was observed for age-matched healthy control subjects.

As the autonomic nervous system controls several systems and pathways in the body, it is recommended to assess its sympathetic and parasympathetic limbs by sequential tests [9]. HRV was measured for beat-to-beat changes in heart rate. This analysis serves as a dynamic window into autonomic function and balance. Assessment of the HRV, LST, CPT, DBT, and VM can reveal early CADs and their contribution primarily to morbidity, mortality, and survival of patients with cancer on chemotherapy. Resting-state parameters such as heart rate (HR), respiratory rate (RR), and blood pressure (BP) were recorded after 10 minutes of supine rest. HR was continuously recorded on an eight-channel digital physiograph machine (Labchart, ADI, Australia) using a lead II electrocardiogram (ECG). The systolic BP response during LST and the diastolic BP response during CPT were used to assess sympathetic reactivity. In the same way, the E: I ratio (expiration to inspiration) and changes in HR during the DBT and the Valsalva ratio (VR) during VM were used as assessment tools for parasympathetic reactivity.

Statistical analyses were performed using SPSS version 20 for Windows (IBM Corp., Armonk, NY, USA) [10]. Gaussian data distribution was checked for all recorded parameters, and appropriate statistical tests were applied. Comparison between the patient and control groups was performed using the Student’s t-test or Mann-Whitney U test as indicated. The level of significance was set to p-values <0.05.

## Results

The mean age of the patient group was 53.94 ± 3.30 years, and the 25 age-matched healthy females that comprised the control group had a mean age of 51.78 ± 4.06 years. The demographic profile and baseline physiological parameters are presented in Table 1. Patients with cancer had significantly higher baseline BP and resting HR and lower body weight.

**Table 1:** Demographic profile and baseline parameters. Values shown are mean ± SD; ^a^ = p-values are significant (p < 0.05).

Baseline parameters	Patients (n = 25)	Controls (n = 25)	P-value
Age (years)	53.94 ± 3.30	51.78 ± 4.06	0.99
Height (cm)	165.8 ± 1.4	166.4 ± 2.1	0.11
Weight (kg)	51.7 ± 1.2	59.9 ± 1.9	0.001^a^
Systolic blood pressure (mmHg)	132 ± 15.82	118.93 ± 13.26	0.047^a^
Diastolic blood pressure (mmHg)	82.53 ± 10.26	77.60 ± 13.22	0.014^a^
Heart rate (per minute)	78.67 ± 11.94	70.60 ± 13.22	0.003^a^
Respiratory rate (per minute)	19.67 ± 5.44	17.82 ± 4.04	0.07

The AFT and HRV parameters obtained for the parasympathetic and sympathetic responses are summarized in Table 2. The results showed that patients with cancer on chemotherapy had significantly lower reactivity parameters than healthy controls. The time domain parameters of HRV were also substantially lower in patients with cancer compared to healthy control subjects. In frequency domain analysis of HRV, only total power (absolute) was considerably reduced in patients.

**Table 2:** Autonomic reactivity and HRV analysis in the patients and controls. Values are shown as median (1 quartile–3 quartiles); ^a^ = p-values are significant (p < 0.05). HR = change in heart rate; E:I = expiration-to-inspiration ratio; VR = Valsalva ratio; ∆SBP = change in systolic blood pressure; ∆DBP = change in diastolic blood pressure; SDNN = standard deviation of the R-R intervals; RMSSD = root mean square of successive RR intervals; NN50 = number of RR intervals equal to or greater than 50 ms; pNN50 = percentage of NN50; SDSD = standard deviation of differences between adjacent RR intervals; Ab = absolute unit for total power of the entire frequency spectrum of heart rate variability

Parameters	Patients (n = 15)	Controls (n = 15)	P-value
SDNN (ms)	12.96 (10.47–34)	35.69 (24.40–42.31)	0.024^a^
RMSSD (ms)	11.54 (8.37–29.95)	35.07 (17.9–43.65)	0.021^a^
pNN50 (%)	0.00 (0.00–4.35)	6.70 (0.31–10.59)	0.023^a^
SDSD	14.30 (8.38–29.91)	35.12 (26.04–43.71)	0.017^a^
Total power (ab)	292.47 (155.57–1116.50)	1289.98 (599.24–2385.37)	0.036^a^
∆HR (bpm)	8 (3–13)	22 (18–25)	0.001^a^
E:I	1.11 (1–1.16)	1.35 (1.23–1.4)	0.001^a^
VR	1.19 (1.03–1.73)	1.72 (1.34–2.14)	0.015^a^
LST ∆SBP (mmHg)	-20 (24–30)	14 (10–20)	0.003^a^
CPT ∆DBP (mmHg)	10 (4–12)	16 (10–20)	0.004^a^

## Discussion

Our study showed that cardiac autonomic functions were significantly affected in patients with breast cancer on chemotherapy. Almost all reactivity parameters were significantly reduced compared to age-matched healthy subjects. There was an increase in supine resting BP and HR in patients with cancer, which can result from increased sympathetic neurovascular tone. It has been suggested that the decrease in absolute power of the lower frequency component is associated with sympathetic activation. The time domain parameter of the parasympathetic component (RMSSD, NN50, pnn50, and SDSD) showed significantly lower values in the patient group. Lower resting parasympathetic tone is an essential feature in the pathophysiology of CAD [2]. In their study, Fagundes et al. (2011) reported similar results of HRV dysfunctions in their research, with HRV as a critical biomarker for cancer-related fatigue [3].

Autonomic reactivity parameters were also significantly decreased in the patient group compared to those of controls, suggesting the involvement of peripheral and central autonomic nerve fibers. This may be because of the chemotherapeutic drugs taken by the patients or the disease itself. Based on the Indian autonomic scoring criteria (Khandelwal et al., 2011) [11], our findings indicated that mild-to-moderate (score = 5-7) autonomic dysfunction was present in the patients’ group.

Teng et al. (2021), in a recent review, also reported that CADs in cancer survivors affect the quality of life and are associated with increased mortality risk [12]. Coumbe et al. (2018) described the lack of data on cancer chemotherapy-induced CAD affecting the survival of patients. The underlying pathophysiology of CAD in carcinoma is not addressed in detail and likely involves disease and its treatment complications [13]. The chemotherapy for breast carcinoma involves doxorubicin, 5-FU, cyclophosphamide, trastuzumab, and paclitaxel. Doxorubicin, cyclophosphamide, and 5-FU have cardiac adverse effects [14]. 5-FU may lead to vasospasm of coronary vessels, leading to acute chest pain following infusion, whereas cyclophosphamide leads to toxic cardiac necrosis at high doses (>200 mg/kg). Doxorubicin has significant cardiac toxicity in short- and long-term use. The acute form is characterized by abnormal ECG, i.e., ST and T-wave alterations with arrhythmia, and is of significant concern [15]. The chronic condition develops owing to cumulative toxicity (total dose >550 mg/m^2^), leading to congestive heart failure with mortality up to 50%. Simultaneously administering other cardiotoxic drugs (cyclophosphamide and trastuzumab) increases the risk of cardiac damage, although the concomitant administration of dexrazoxane may reduce the toxicity [14]. Careful detection of autonomic dysfunction in patients with breast cancer is critical, as it empowers clinicians to stratify risk factors and offer better management of the patients on chemotherapy.

The present study has some limitations. The number of patients with cancer was small, and the patients were recruited from a single referral health care center.

## Conclusions

In the present study, the sympathetic and parasympathetic parameters showed a significant loss of autonomic functions in the patient group compared to the healthy controls. This may be because of the chemotherapeutic drugs taken by the patients or the disease itself. Because CAD is highly prevalent in patients with breast cancer and is associated with various symptoms and shorter life expectancy, it is essential to study CAD in the cancer population. Our study showed that HRV and Ewing’s battery of tests is a reliable diagnostic and prognostic method for detecting and scoring CAD in patients with breast cancer. Other prospective studies are needed to establish the relationship between CAD in patients with cancer and chemotherapy.
